# Letter from the Editor in Chief

**DOI:** 10.19102/icrm.2021.120504

**Published:** 2021-05-15

**Authors:** Moussa Mansour


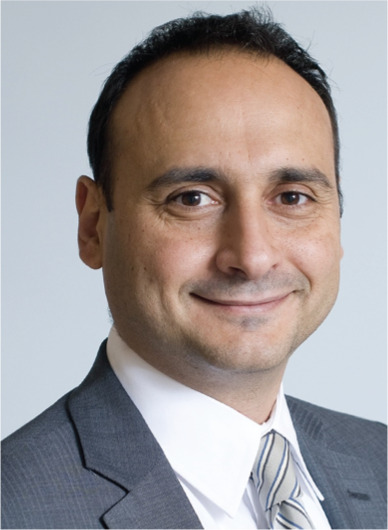


Dear Readers,

Atrial fibrillation (AF) affects a large number of people in the United States and represents a major clinical and economic burden. One of the major complications of AF is embolic stroke and the subsequent increased morbidity and mortality rates associated with it. Data guiding the treatment of AF, specifically concerning stroke prevention, are robust, and a large number of pharmacological and nonpharmacological therapies that have proven to be effective may be used once AF has been detected. Nevertheless, AF can be asymptomatic, and it is not uncommon for stroke to be the first presentation of this condition. As a result, early detection of AF is critical to prevent its downstream complications.

In the past few years, interest has grown in population-based screening for AF. However, a definitive study yielding clear-cut endorsements for screening for AF has not yet been completed. As a result, detailed recommendations regarding when, who, and how to screen for AF remain elusive.

Numerous studies are currently being conducted with the aim of demonstrating the value of population-based screening. One such study is the STROKESTOP (NCT01593553) trial,^1^ which was discussed during the late-breaking clinical trials session of the recent 2021 European Heart Rhythm Association virtual meeting. This study aimed to compare outcomes between more than 13,000 people aged 75 or 76 years who were invited to undergo screening for AF and a similar number of people who were not invited for screening in a randomized fashion. Screening consisted of intermittent ambulatory electrocardiogram recordings performed twice daily for two weeks and, when detected, AF was treated accordingly with oral anticoagulation. The follow-up period was five years, and the primary endpoint was the occurrence of ischemic or hemorrhagic stroke, systemic embolism, major bleeding leading to hospitalization, or death from any cause. Although only half of the people invited to participate in screening ended up doing so, the study still demonstrated a small but statistically significant beneficial outcome in the screening group (hazard ratio: 0.96; P = 0.045).

STROKESTOP is an important study that may help to fill in the knowledge gap concerning screening for AF. Other similar studies are also currently underway and will likely not only demonstrate the value of screening but also provide clear-cut recommendations about who and when to screen. Moreover, the widespread use of consumer devices with the capability to detect AF may facilitate this task and make it easily accessible.

Best wishes for a joyful spring and I hope that you find the content of this issue of *The Journal of Innovations in Cardiac Rhythm Management* of educational value.

Sincerely,


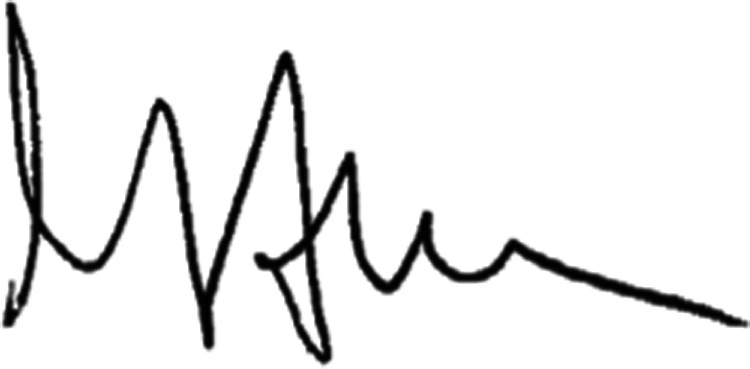


Moussa Mansour, MD, FHRS, FACC

Editor in Chief

*The Journal of Innovations in Cardiac Rhythm Management*

MMansour@InnovationsInCRM.com

Director, Atrial Fibrillation Program

Jeremy Ruskin and Dan Starks Endowed Chair in Cardiology

Massachusetts General Hospital

Boston, MA 02114
